# Gastric and urinary bladder pressures correlate with intra-abdominal pressure in patients with morbid obesity

**DOI:** 10.1007/s10877-021-00728-7

**Published:** 2021-06-17

**Authors:** Shadi Hamoud, Siham Abdelgani, Michal Mekel, Safa Kinaneh, Ahmad Mahajna

**Affiliations:** 1 Department of Internal Medicine E, Rambam Health Care Campus, Haifa, Israel; 2 Ruth & Bruce Rappaport Faculty of Medicine, Technion– Israel Institute of Technology, Haifa, Israel; 3 Department of Internal Medicine A, Rambam Health Care Campus, Haifa, Israel; 4 Department of General Surgery, Rambam Health Care Campus, POB 9602, 3109601 Haifa, Israel; 5 Department of Physiology, The Ruth & Bruce Rappaport Faculty of Medicine, Technion– Israel Institute of Technology, Haifa, Israel

**Keywords:** Intra-abdominal pressure, Gastric pressure, Urinary bladder pressure, Laparoscopy

## Abstract

Intra-abdominal pressure (IAP) affects cardio-respiratory and hemodynamic parameters and can be measured directly or indirectly by measuring gastric or urinary bladder pressure. The aim of this study was to investigate the correlation between IAP, gastric pressure and urinary bladder pressure in patients with morbid obesity, at normal and elevated levels of IAP in two positions. As well, to examine the effects of increasing IAP and patient's position on hemodynamic and respiratory parameters. Twelve patients undergoing laparoscopic bariatric surgery were included. IAP, gastric pressure, and urinary bladder pressure were measured while patients were in the supine position and after 45° anti-Trendelenburg tilt. Mean inspiratory pressure, peak inspiratory pressure, and tidal volume were recorded and assessed. In supine position; directly measured IAP was 9.1 ± 1.8 mmHg, compared to 10 ± 3.6 and 8.9 ± 2.9 mmHg in the stomach and bladder, respectively. Increasing IAP to 15 mmHg resulted in an increased gastric pressure of 17 ± 3.8 mmHg, and urinary bladder pressure of 14.8 ± 3.9 mmHg. Gastric and urinary bladder pressures strongly correlated with IAP (R = 0.875 and 0.847, respectively). With 45° anti-Trendelenburg tilt; directly measured IAP was 9.4 ± 2.2 mmHg, and pressures of 10.8 ± 3.8 mmHg and 9.2 ± 3.8 mmHg were measured in the stomach and the bladder, respectively. Increasing IAP to 15 mmHg resulted in elevating gastric and bladder pressures to 16.6 ± 5.3 and 13.3 ± 4 mmHg, respectively. Gastric and urinary bladder pressures had good correlation with IAP (R = 0.843 and 0.819, respectively). Changing patient position from supine to 45° anti-Trendelenburg position resulted in decreased mean and peak inspiratory pressures, and increased tidal volume. Basal IAP is high in patients with morbid obesity. IAP shows positive correlation to gastric and urinary bladder pressures at both normal and elevated levels of IAP. Anti-Trendelenburg tilt of mechanically ventilated morbidly obese patients resulted in favorable effects on respiratory parameters.

**Trial Registration**: The study was retrospectively registered in the NIH registry. Registration number is pending.

## Background

Increased intra-abdominal pressure is a common clinical condition that can lead to severe morbidity if continued without appropriate treatment. Abdominal compartment syndrome (ACS) is a serious complication that can cause multi-organ failure (MOF) and death. ACS is defined as a sustained IAP > 20 mmHg (with or without an abdominal perfusion pressure < 60 mmHg) that is associated with new organ dysfunction or failure [1].

There has been a marked improvement in management, diagnosis and treatment of intra-abdominal hypertension (IAH) and ACS based on The consensus definitions of the World Society of the Abdominal Compartment Syndrome (WSACS) that were published in 2006 [1, 2] followed by the clinical practice guidelines published in 2007 [3] and updated in 2013 [4]. In addition, many surveys were conducted to determine the current state of awareness and knowledge of medical stuffs and the use of evidence-based medicine regarding IAH and ACS [5, 6].

ACS can be primary or secondary depending on whether the etiology is related to the abdominal-pelvic cavity and requiring specific intervention of a target organ in case of primary while in secondary ACS there is no abdominal disease that requires specific surgical correction, but the high abdominal pressure is associated with an organic dysfunction that requires immediate surgical decompression. Common causes to ACS include: abdominal or pelvic trauma, intra-abdominal hemorrhage, retroperitoneal hematoma or edema. Other conditions as bowel obstruction, ascites, and necrotizing pancreatitis may lead to ACS as well [7–10].

Sustained IAP results in elevated intrathoracic pressure which compromises pulmonary function, dynamics, increases afterload, decreased venous return and cardiac output. Perfusion to the kidneys and intestinal mucosa is severly reduced [11–14]

Early diagnosis and treatment of IAH is essential to avoid MOF and death. There is still controversy regarding the ideal method for measuring IAP. A variety of studies suggest to frequently measure IAP in critically il patients. Current practice assesses IAP indirectly through the measurement of intra vesicular pressure, however, only few studies have been performed to establish the actual relationship between IAP and urinary bladder pressure (UBP) [15–17]. In obese patients no studies have validated this technique.

The aims of this study were to asses the basal IAP and to investigate the correlation between IAP, gastric pressure (GP) and urinary bladder pressure (UBP), in patients with morbid obesity (body mass index- BMI > 40 kg/m^2^) who underwent bariatric surgery. The measurements were performed at normal and elevated levels of IAP in two positions: supine and 45 degrees anti-trendelenburg tilt. The effect of increasing IAP and change in the patient position on hemodynamic parameters (mean arterial pressure and heart rate) and respiratory parameters (mean inspiratory pressure, peak inspiratory pressure and tidal volume) were evaluated as well.

## Methods

### Patients

Male and female patients aged ≥ 18 years with morbid obesity were included in the study, while undergoing bariatric surgery in the Department of General Surgery at Rambam Health Care Campus (Rambam). Main exclusion criteria were any contraindications to laparoscopic surgery or urethral catheterization; known intra-abdominal adhesions or ventral hernia due to previous surgery; chronic obstructive pulmonary disease (PaCo_2_ > 50 mmHg, FEV_1_ < 1 L); or marked left ventricular dysfunction (left ventricular ejection fraction < 25%).

### Experimental protocol

The study was approved by Rambam's ethics committee (ethics number: 0019–12), and consent was obtained from all the participants. After general anesthesia was induced, a nasogastric tube and urinary Foley catheter were inserted. The study was conducted in two stages: (1) while patients were in the supine position, IAP was measured and adjusted for 5 min, and then (2) IAP was increased to 15 mmHg by insufflation of CO2 for 5 min. Gastric and urinary bladder pressures were measured together with hemodynamic and respiratory parameters at each stage. Subsequently, the patient was up-tilted to 45° anti-Trendelenburg position and pressure and hemodynamic measurements were repeated.

#### Measurement of intra-abdominal pressure

The pressure within the abdominal cavity was measured directly using an automatic CO2 insufflation measurement device (KARL STORZ Endoskope 264,320 20, Tuttlingen, Germany).

#### Gastric pressure

Following insertion of the nasogastric tube, the stomach was drained and filled with 50 ml of normal saline. A rigid pressure tube was connected to the gastric tube using a male to male connector at one end, and to the monitor through a pressure transducer at the other end. The system was flushed with normal saline and pressure transducer zeroed at the mid axillary line.

### Urinary bladder pressure

A Foley catheter was placed into the urinary bladder. The bladder was drained and filled with 50 ml of sterile normal saline. The drainage tube was clamped just beyond the aspiration port, and a 16-gauge needle connected to the rigid pressure tube was inserted into the port. The tube was connected to the monitor by a pressure transducer. The system was flushed with normal saline and the pressure transducer zeroed using the symphysis pubis as the zero reference point.

### Statistical analysis

Sample size calculation was based on the http://biomath.info/power/prt.htm for paired samples. We assumed that the mean difference between the two measurements will be 2 units with 2.5 SD. In this case we needed to recruit less than 15 patients. Variables are presented as mean and standard deviation. Statistical analysis included the χ^2^ test for categorial variables and Student t-test for continuous variables. P-value < 0.05 was considered statistically significant.

## Results

Of 13 patients included in the study, one was subsequently excluded due to a huge ventral hernia after previous open cholecystectomy. Table 1 summarizes participants’ characteristics. There were eight females and four males with an average age of 35 ± 11 years, and average BMI of 43 ± 5.8 kg/m^2^. The American Society of Anaesthesiologist (ASA) physical status score was II for eight patients and III for four patients.

**Table 1:** Characteristics of patients

	n = 12	Range
Males	4	
Females	8	
Average age (years, range)	35 ± 12	18–55
Weight (kilograms)	120 ± 20	102–150
Height (meters)	1.66 ± 0.9	1.54–1.80
BMI (kg/m^2^)	43 ± 5.8	37–55

### Correlation between directly measured IAP, GP and UBP

**Fig. 1:**
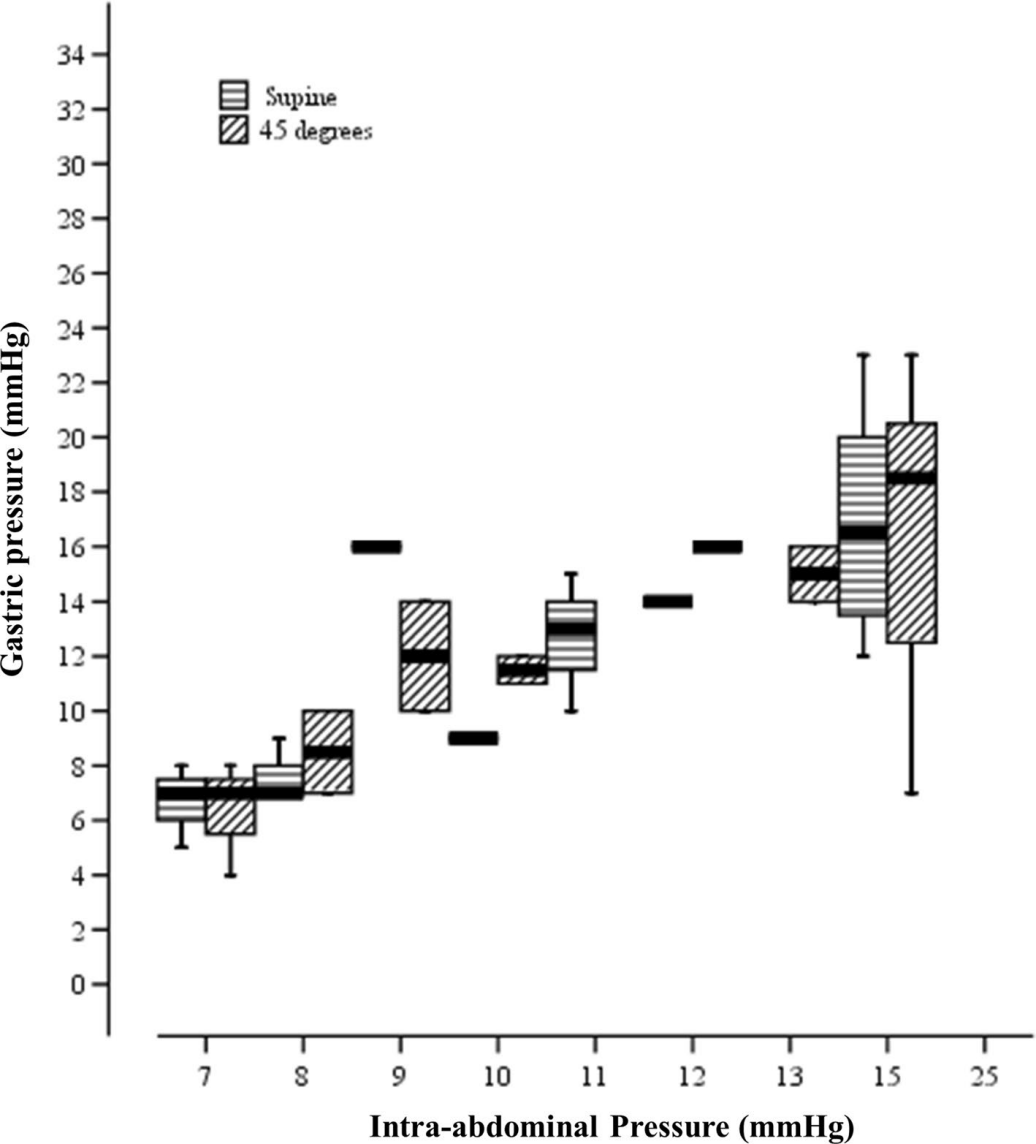
Correlation between directly measured Intra-abdominal Pressure (mmHg) and Gastric Pressure (mmHg) in two positions; Supine and 45° anti-Trendelenburg position. Values are presented as mean ± SD

**Fig. 2:**
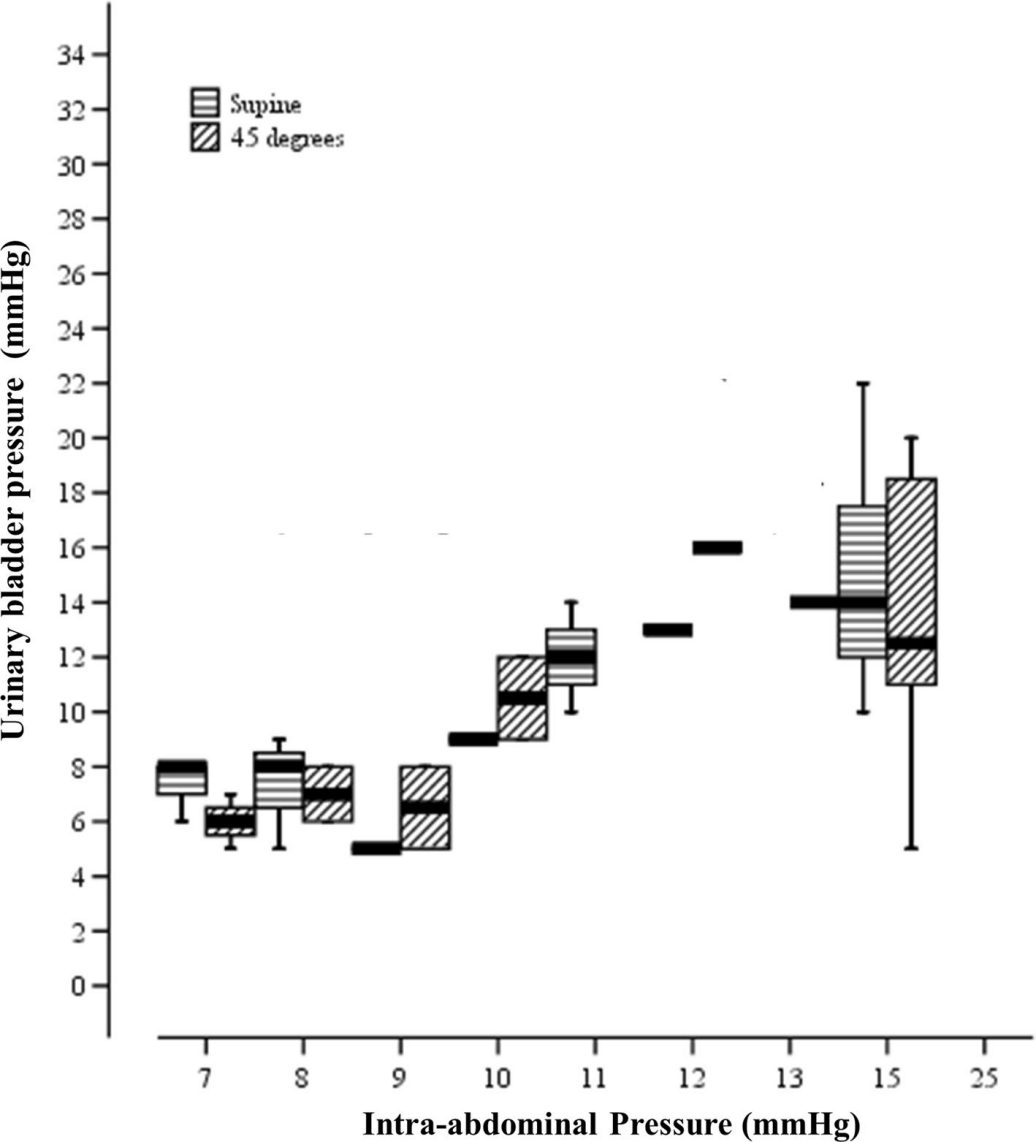
Correlation between directly measured Intra-abdominal Pressure (mmHg) and Urinary Bladder Pressure (mmHg) in two positions; Supine and 45° anti-Trendelenburg position. Values are presented as mean ± SD

When subjects were in the supine position; the directly measured IAP was 9.1 ± 1.8 mmHg, compared to pressures of 10 ± 3.6 mmHg and 8.9 ± 2.9 mmHg in the stomach and urinary bladder, respectively. Increasing IAP to 15 mmHg resulted in an increased GP of 17 ± 3.8 mmHg, and increased UBP to 14.8 ± 3.9 mmHg (Figs. 1 and 2). The correlation coefficients of GP and UBP to IAP were 0.875 and 0.847, respectively (Table 2). After elevating patients to 45 degrees anti-Trendelenburg position, direct IAP increased to 9.4 ± 2.2, compared to 10.8 ± 3.8 and 9.2 ± 3.8 mmHg in the stomach and the urinary bladder, respectively. Increasing IAP to 15 mmHg resulted in an increased GP of 16.6 ± 5.3 mmHg, and of the UBP to 13.3 ± 4.9 mmHg, while GP and UBP strongly correlated with IAP (R = 0.843 and 0.819, respectively) (Table 2).

**Table 2:** Correlation between IAP, GP and UBP in supine and with 45 degree anti-trendelenburg elevation, in baseline and with wlwvated IAP to 15 mmHg

	Position	Mean (SD) by group of IAP and significance of position's diff				p-value (IAP: baseline Vs 15 mmHg)	Correlation with IAP (all groups)	
	Supine	9.1 (1.8)	p- value^a^	15.0 (0.00)	p- value^b^	NA	R	p-value
IAP	45 Degr	9.4 (2.2)		15.0 (0.00)		NA		
GI	Supine	10.0 (3.6)		17.00 (3.8)		< 0.001	0.875	< 0.001
	45 Degr	10.8 (3.8)	0.713	16.6 (5.3)	0.531	< 0.001	0.843	< 0.001
Bladder	Supine	8.9 (2.9)		14.8 (3.9)		< 0.001	0.847	< 0.001
	45 Degr	9.2 (3.8)	0.686	13.3 (4.9)	0.326	< 0.001	0.819	< 0.001
MAP	Supine	87.0 (11)		104.0 (11.0)		< 0.01	0.634	< 0.001
	45 Degr	99.0 (20.0)	0.760	106.0 (14.0)	0.695	0.33	0.291	0.085
HR	Supine	75 (13)		75 (16)		0.71	0.184	0.284
	45 Degr	78 (17)	0.718	81 (17)	0.181	0.25	0.152	0.377
MIP	Supine	11 (2)		13 (3)		< 0.001	0.407	0.014
	45 Degr	10 (2)	0.788	12 (3)	**0.021**	< 0.001	0.447	0.006
PIP	Supine	27 (1)		31 (2)		< 0.005	0.777	< 0.001
	45 Degr	24 (3)	0.792	28 (3)	**0.004**	< 0.001	0.748	< 0.001
Tv	Supine	0.63 (0.1)		0.52 (0.1)		0.01	-0.495	0.002
	45 Degr	0.65 (0.1)	0.770	0.62 (0.1)	**0.016**	0.130	-0.366	0.028

*p* < 0.05 is considered statistically significant

^a^By comparing of correlations with IAP

^b^By Wilcoxon Signed Ranks Test

### Effect of increased IAP on mean arterial pressure (MAP) and heart rate (HR)

In the supine position, increasing the IAP to 15 mmHg resulted in an elevation of the MAP from 87 ± 11 mmHg to 104 ± 11 mmHg (p < 0.01). After 45° anti-Trendelenburg tilt, a similar increase of IAP resulted in an elevated MAP from 99 ± 20 mmHg to 106 ± 14 mmHg (p = 0.33, Table 2). In the supine position, increasing IAP to 15 mmHg caused no change in the HR (75 ± 13 bpm and 75 ± 16 bpm, p = 0.71). Also after 45^o^ anti-Trendelenburg tilt, a similar increase of IAP resulted in non-significant elevation of the HR from 78 ± 17 bpm to 81 ± 17 bpm (p = 0.25, Table 2).

### Effect of increased IAP on mean inspiratory pressure (MIP), peak inspiratory pressure (PIP) and Vt

In the supine position, increasing IAP to 15 mmHg resulted in MIP elevation from 11 ± 2 mmHg to 13 ± 3 mmHg (p < 0.001). After elevating the patients to the 45° anti-Trendelenburg position, a similar increase of the IAP resulted in MIP elevation from 10 ± 2.4 mmHg to 12 ± 3 mmHg (p < 0.001, Table 2). Similarly, in the supine position, increasing the IAP to 15 mmHg resulted in an increase of PIP from 27 ± 1 mmHg to 31 ± 2 mmHg (p < 0.005). After tilting patients to 45° anti-Trendelenburg position, a similar IAP increase resulted in PIP elevation from 24 ± 3 mmHg to 28 ± 3 mmHg (p < 0.001, Table 2).

In the supine position, increasing IAP to 15 mmHg resulted in a decreased Vt from 0.60 ± 0.1 lit to 0.52 ± 0.1 lit (p = 0.01). After a tilt to 45° anti-Trendelenburg position, a similar increase of the IAP decreased the Vt from 0.65 ± 1 lit to 0.62 ± 1 lit (p = 0.13, Table 2). While patients were with IAP of 15 mmHg, changing the patient position from supine to the 45° anti-Trendelenburg position resulted in decreased MIP (from 13 ± 3 to 12 ± 3 mmHg, p = 0.021) and PIP (from 31.2 ± 2.3 to 28.1 ± 3.3 mmHg, p = 0.004), and increased Vt (from 0.52 ± 0.1 to 0.62 ± 1 lit, p = 0.016).

## Discussion

Morbid obesity has been proclaimed by the WHO Statement as the epidemic of the 21st Century [18, 19], and is associated with considerable morbidity and mortality. With the widespread success of damage control laparotomy, ACS has become a virtual epidemic in trauma centers throughout the world [20–22]. Increasing numbers of critically ill patients are obese; therefore the special consideration of IAP and ACS in this patient population has become significantly more relevant.

There is an exponential increase in studies on IAP and ACS in non-obese patients in the literature, but very few studies include the measurement of IAP in obese patients.

The diagnosis of IAH or ACS is heavily dependent on the reproducibility of the IAP measurement technique. Over time, literature has suggested many methods to assess IAP. The ideal tool is still controversial [15]. Malbrain MLNG et al. showed in there study a poor correlation between IAP and abdominal perimeter [23]. Other studies have shown that a clinical estimation of IAP examiner’s feeling of the tenseness of the abdomen is not accurate, with a low sensitivity [24].

Consequently, the IAP needs to be measured with a more accurate and reliable tool. Different direct and indirect measurement methods have been reported [25, 26]. Traditionally, the urinary bladder pressure has been used as the method of choice for measuring the IAP. The technique was originally described by Kron et al. [8]. It is safe, minimally invasive, and has minimal side effects and complications.

Another useful site for measuring IAP is in the stomach through a nasogastric tube, which can be used when the patient has no Foley catheter in place, or when bladder pressure measurement is not possible due to absence of free contractibility of the bladder wall [27]. GP measurement is cheap, does not interfere with urine output, and has no risk of infection. Both methods are ideal for screening and monitoring of critically ill patients.

Several studies have addressed the validation of indirect versus direct measurements of the IAP during laparoscopy in non-obese patients. Yol et al. compared bladder pressure with direct insufflation pressure during laparoscopic cholecystectomy in 40 patients, and obtained a positive correlation between the two measurements (R = 0.973, P < 0.0001) [28]. Likewise, Fusco et al. compared direct laparoscopic insufflation pressure with bladder pressure in patients undergoing laparoscopy, and demonstrated a good correlation in pressure values across the IAP range from 0 to 25 mmHg between the direct and indirect measurement methods [29]. There have been no studies comparing the direct IAP with urinary bladder or gastric pressures in patients with morbid obesity.

In the current study, we found that the baseline IAP measured in the urinary bladder and stomach of patients with morbid obesity was higher than values reported in non-obese subjects, consistent with the literature. Sugerman et al. showed that the urinary bladder pressure was significantly higher in obese compared to non-obese patients (18 ± 0.7 vs. 7 ± 1.5 cmH_2_O, respectively), and concluded that increased IAP, as reflected in urinary bladder pressure, contributes to health risks associated with severe obesity [30]. Similarly, Lambert et al. concluded that IAP is elevated in patients with morbid obesity, and increased IAP is a function of central obesity associated with increased morbidity [31]. In our study, we included twice female patients than males. Although female obesity is different from male obesity (abdominal impact), our results demonstrated similar behavior of IAP when comparing male and female patients, thus one can exclude impact of gender on IAP- neither directly measured nor indirectly (GP or UBP). Moreover, our results are in accordance with the study of Smit et al. that had demonstrated a direct correlation between BMI and IAP, whereas correlation between IAP and indices of central obesity were not significant [32].

The chronic elevation of IAP can explain why many severely obese patients, especially those with sleep apnea and hypoventilation, have found they must sleep in the sitting position, presumably to lower the effects of increased IAP on their thoracic cavity. In our study, we found that changing the patients’ position from supine to 45° anti-Trendelenburg elevated position decreased the inspiratory pressures and increased Vt, findings that can be translated to a decrease in respiratory effort. Furthermore, increasing IAP from baseline to 15 mmHg resulted in a rise in the MAP with no change in the HR, both in the supine position and after a tilt of 45° anti-Trendelenburg. Various mechanisms, such as venous compression caused by elevated IAP (with compression of the abdominal vasculature and organs), and the pharmacological action of the absorbed CO_2_, have been proposed to explain these transient adverse hemodynamic effects [33, 34]. It has also been shown that increased IAP during carbon dioxide pneumoperitoneum is associated with increased mean arterial blood pressure and systemic vascular resistance [35, 36].

In accordance with Lambert et al. who demonstrated elevated IAP in patients with morbid obesity compared to non-obese patients [31], we also found that baseline IAP is high in patients with morbid obesity, and obtained a good correlation between the direct IAP measured by laparoscopic insufflation route on the one hand, and urinary bladder and gastric pressures measured in morbidly obese patients on the other hand, both at normal and elevated levels of IAP. In addition, we found that changing the patients' position from the supine position to 45° anti-Trendelenburg elevation position caused decreased mean and peak inspiratory pressures, while increasing Vt. Therefore, patients with increased IAP are expected to benefit from the anti-Trendelenburg position as it improves respiratory parameters without hemodynamic compromise.

## Conclusions

Our study demonstrated that in obese patients there is good correlation between directly measured IAP and indirectly measured pressures through the stomach or the urinary bladder. Changing the position of the patient with increased IAP to the anti-Trendelenburg position is helpful in improving respiratory parameters without interference to the patient's hemodynamic status. Small number of participants is a limitation in our study, though the consistent results in all the participants allows us to apply our results to the obese patioents population. Future studies with more participants would strengthen our findings.

## Data Availability

All data generated or analysed during this study are included in this published article.

## Abbreviations

ACS: Abdominal compartment syndrome
ASA: American Society of Anaesthesiologist
GP: Gastric pressure
HR: Heart rate
IAH: Intra-abdominal hypertension
IAP: Intra-abdominal pressure
MAP: Mean arterial pressure
MIP: Mean inspiratory pressure
PIP: Peak inspiratory pressure
Vt: Tidal volume
WSACS: World Society of the Abdominal Compartment Syndrome
